# Experimental Investigation on Rotating Electrochemical Etching of a Micro Spiral Cylindrical Electrode

**DOI:** 10.3390/mi10100704

**Published:** 2019-10-16

**Authors:** Qiuju Xiong, Huali Wang, Xueliang Wang, Shihui Deng, Yong Liu, Zhen Lv

**Affiliations:** 1 Medical Device Department, Shandong Drug and Food Vocational College, Weihai 264210, China; 100298@sddfvc.cn (Q.X.); wanghuali@ustc.edu (H.W.); 110156@sddfvc.cn (X.W.); 2 Associated Engineering Research Center of Mechanics and Mechatronic Equipment, Shandong University, Weihai 264209, China; 201614777@mail.sdu.edu.cn (S.D.); 201916559@mail.sdu.edu.cn (Z.L.)

**Keywords:** rotating electrochemical etching, spiral vortex flow, electropolishing, spiral electrode, thread structure

## Abstract

To realize the electrochemical etching of a micro spiral cylindrical electrode, a new method of rotating electrochemical etching is proposed, and its process is further studied. First, according to the electrochemical etching principle, the machining mechanism of rotating electrochemical etching of a micro spiral cylindrical electrode is introduced. Second, based on the spiral vortex theory in the Taylor-Couette system, the effect of the high-speed rotating cylindrical microelectrode on its external flow field is analyzed. Third, the effects of rotation direction, rotation speed, machining voltage, and machining time on the thread structure are analyzed by experiments. Finally, a spiral cylindrical microelectrode with good surface thread shape is fabricated within two minutes by using the optimized machining parameters. It is proved that the rotating electrochemical etching method is an easy way to fabricate a micro spiral cylindrical electrode with high efficiency and low cost.

## 1. Introduction 

With the combination of numerical control technology and micromachining, the tool microelectrodes in electrochemical micromachining (EMM) and micro electrochemical discharge machining (micro-ECDM) play the same role as those in traditional machining. The shape, size, and accuracy of tool electrodes have great influence on the quality and accuracy of micromachining. The fabrication of microelectrodes has always been a research hotspot in the field of micromachining [1,2].

As tool electrodes in EMM and micro-ECDM, the fabrication of microelectrodes has been studied extensively, and the single-step cylindrical electrode is the simplest and most commonly used tool electrode. However, with the improvement in machining ability and the reduction in machining size, the single-step cylindrical electrode cannot meet the machining requirements of high aspect ratio and high precision dimensions because of its poor chip removal effect, low processing efficiency, and difficulty in achieving high aspect ratio. Therefore, many scholars have fabricated specially shaped tools, such as a disk microelectrode, micro spherical electrode, multi-stepped cylindrical microelectrode, array electrodes, spiral microelectrode, etc., based on cylindrical electrodes, which can improve the machining accuracy and efficiency. V. Rathod used the reverse-reaction electrochemical machining to fabricate a disk-shaped electrode with the disk diameter of ∅ 200 μm, disk thickness of 70 μm, and shank diameter of ∅ 84 μm [3]. Wang fabricated disk microelectrodes by wire cut electrochemical discharge machining (WECDM) and EMM, whose disk diameter, shank diameter, disk thickness, and shank length are ∅ 266 μm, ∅ 127 μm, 329 μm, and 1200 μm, and the small tapered micro-holes with the aspect ratio of 12.3 were fabricated through these microelectrodes [4,5]. Liu used one pulse electro discharge to melt the tip of the multi-stepped cylindrical electrode to form a spherical end with the spherical diameter of ∅ 10 μm and the shank diameter of ∅ 6 μm [1,2]. Deng efficiently fabricated the three-stepped cylindrical microelectrodes with a diameter of ∅ 15 μm, length of 1500 μm, and coaxiality error of less than 1 μm by rotating electrochemical etching [6]. Sheu fabricated arrayed spherical microelectrodes by WEDM and one pulse electro discharge, with the spherical tip diameter of ∅ 15 μm and the shank diameter of ∅ 10 μm [7,8]. Kim fabricated multiple cylindrical microelectrodes with a diameter of ∅ 35 μm using reverse EDM [9]. However, there are few studies on spiral microelectrodes, and the main difficulty in machining spiral microelectrode fabricating a spiral groove on the surface of the cylindrical microelectrode.

Among the above specially shaped microelectrodes, the shank diameters of the disk microelectrode and the spherical microelectrode are smaller, and the stray corrosion on the machined sidewall is less, which can improve the machining precision to a certain extent. The aspect ratio of the multi-stepped cylindrical microelectrode is relatively large, and the diameter of the terminal electrode can be machined to several microns stably, which can meet some micro size machining. Array microelectrodes can be used to machine multiple micro holes at one time to improve machining efficiency. However, when the size of the machining gap is less than tens of microns, the machining process cannot proceed smoothly because the machining products cannot be discharged in time [10]. The spiral cylindrical microelectrode, because of its unique spiral groove structure, can take away the electrolysis products generated during the machining process and promote the electrolyte renewal, which is used more and more in the EMM process [11,12]. However, the spiral cylindrical microelectrodes used today are mechanically machined by high-precision Computer Numerical Control (CNC) machine tools, which are expensive, have low machining efficiency, and are not easily machined to a small size due to cutting force. Electrochemical etching, which has no cutting stress, high machining efficiency, and where the smallest microelectrode can be machined to nanosize, is an ideal method for fabricating electrodes [13].

In this paper, based on the conventional EMM process and the effect of electrode rotation on the flow field and diffusion layer around the electrode surface, a new method for machining spiral cylindrical microelectrodes with high machining efficiency, low machining cost, and simple operation is proposed, and the shape and surface quality of the machined microelectrode are good.

## 2. Analysis of Machining Mechanism

### 2.1. Machining Principle

**Figure 1:**
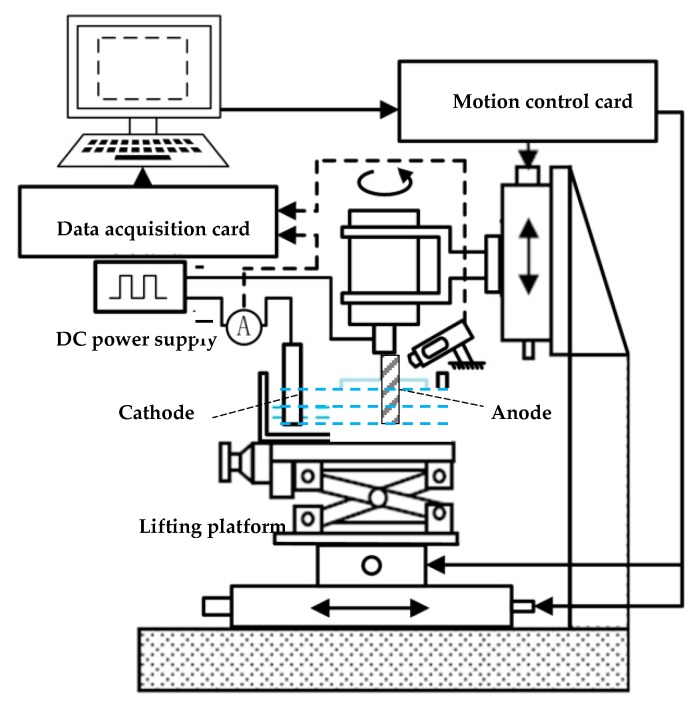
Structure of machine tool.

The spiral cylindrical microelectrode was fabricated by the rotating electrochemical etching. The cathode was a stainless steel sheet that was placed horizontally in the electrolytic tank. The fabricated anode electrode was a ∅ 500 μm straightened tungsten wire with higher rotation speed. The electrolyte was 15% NaOH solution, and DC power supply provided energy. The machine tool structure is shown in Figure 1. The ∅ 500 μm tungsten wire was clamped at the lower end of the high-speed motorized spindle through an elastic clamp after the tungsten wire was straightened. The control system of the machine tool maintained a certain machining gap (5 mm) between the anode tungsten wire and the cathode and immersed it in the electrolyte to a certain machining depth. The motorized spindle drove the anode to rotate at a higher rotation speed.

The external diffusion layer of the electrode directly affects the etching rate of electrode [2]. Therefore, the diffusion layer distribution around the electrode surface is the main factor affecting the electrode shape. Rotating electrochemical etching was originally used to fabricate cylindrical microelectrodes with high rotation accuracy, and the rotation speed of the electrode was less than 2000 rpm. Due to the small diameter of the microelectrode, the disturbance to the external fluid, and the diffusion layer was weak. Under a suitable machining voltage, the diffusion layer is uniform. Therefore, the etching rate on the outer surface of the electrode is the same, and the shape of the fabricated microelectrode is cylindrical [13]. However, in the process of deep research on the rotating electrochemical etching, it was found that when the electrode rotation speed was more than 2000 rpm, the fabricated electrode was not uniformly cylindrical, and the electrode surface became uneven, and at higher processing voltages and processing times, a regular spiral groove structure appears on the surface of the electrode, from which can be inferred that the outer diffusion layer of the electrode is spirally distributed, so that the etching rates of each part of the electrode surface are inconsistent.

In the process of the fabricated cylindrical electrode by electrochemical etching, the surface of the anode electrode is etched, and the electrode surface has no metallic luster. However, the surface brightness of the spiral cylindrical electrode fabricated at higher voltage and rotation speed is higher than that of the ordinary cylindrical electrode. Therefore, it can be determined that the surface of the spiral cylindrical microelectrode not only undergoes electrochemical etching but also undergoes electrochemical polishing during the forming process of the spiral groove structure. 

The electrochemical reactions of the anode: W + 8OH^−^→ ${\mathrm{WO}}_{4}^{2-}$ + 4H_2_O + 6e^−^

Electrochemical reaction of the cathode: 6H_2_O + 6e^−^ → 3H_2_↑ + 6OH^−^

The total reaction: W + 2OH^−^ + 2H_2_O → ${\mathrm{WO}}_{4}^{2-}$ + 3H_2_↑

According to the above analysis, the surface of the electrode is electrolytically polished during the machining process of the spiral micro electrode, and the outer diffusion layer of the electrode is spiral upward. In addition, the flow direction of the flow field directly affects the distribution of the diffusion layer, so it can be considered that the external flow field distribution affects the shape of the electrode.

### 2.2. Flow Field Analysis of Electrode Surface

In the process of machining spiral microelectrode by rotating electrochemical etching, the flow field around the electrode surface directly affects the distribution of the diffusion layer, and then the diffusion layer affects the etching rate and electrode shape. However, in this paper, a spiral groove machined on the external surface of cylindrical microelectrode indicated that the etching rate difference occurred on the surface of the electrode, and the distribution of the diffusion layer around the surface of the electrode was consistent with the direction in which the spiral groove extended and the external flow field of electrode changed. 

Few scholars have studied the flow field distribution on the surface of high-speed rotating cylindrical electrodes. However, many scholars have studied the flow field distribution in the Taylor-Couette system. The Taylor-Couette system consists of two concentric rotating cylinders with different diameters and liquid between the cylinder walls. It is found that when the inner and outer cylinders rotate with a certain relative rotational speed difference, the flow field between the inner and outer cylinder walls becomes unstable, and under the action of centrifugal force, an annular Taylor vortex which is equidistantly distributed along the axial direction is formed. And the Taylor vortex flow (TVF) is a steady laminar flow. When the relative rotation speed difference is larger, the TVF becomes unstable and axial fluctuation occurs, and the annular vortex is inclined with respect to the horizontal direction. At this time, the Taylor vortex becomes spiral vortex flow (SVF) [14,15,16].

**Figure 2:**
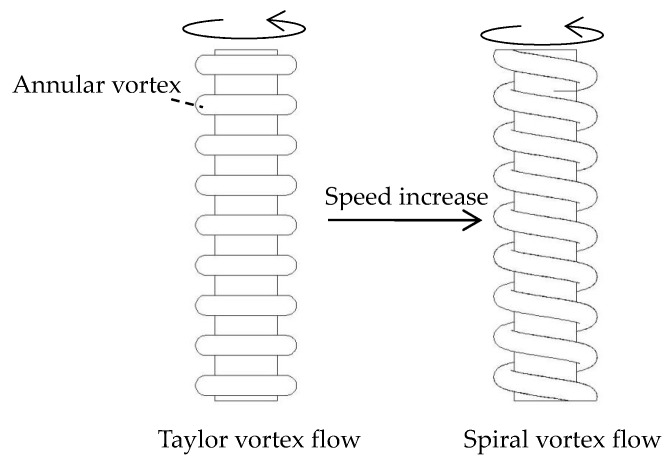
Schematic diagram of from Taylor vortex flow (TVF) to spiral vortex flow (SVF).

Figure 2 shows the pattern of TVF and SVF. The annular vortex is the main flow area, in which the current velocity of the liquid is faster. It can be concluded from the above analysis that the flow pattern of the liquid on the electrode surface is similar to that of the spiral vortex due to the high-speed rotating cylindrical tungsten wire during the machining process of the spiral cylindrical microelectrode. The distribution of diffusion layer changes with the flow of liquid, therefore, the distribution of the flow field is the main factor affecting the shape of the microelectrode.

**Figure 3:**
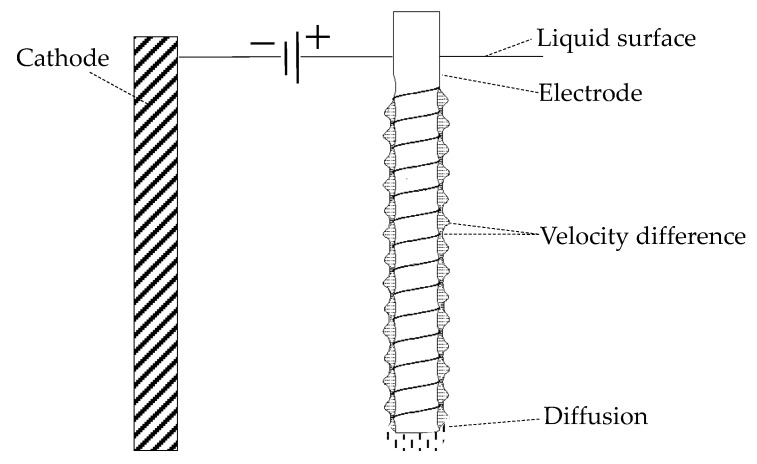
Machining schematic diagram of the spiral cylindrical electrode.

The velocity of fluid flow inside the annular vortex flow is faster, so the electrolyte inside the diffusion layer affected by the vortex flow renews faster, which accelerates the etching rate of electrode material, the electrolyte at the junction of the vortex flow renews slower, and the etching rate of the electrode is slower. In a certain range of current density, the larger etching rate causes the spiral groove on the surface of the cylindrical electrode. The distribution of the diffusion layer around the electrode surface is shown in Figure 3.

Figure 3 shows that the SVF makes the diffusion layer spirally distribute around the microelectrode, the velocity of liquid flow inside the vortex flow is faster, so the etching rate on the surface of the electrode covered by the eddy current is faster, and the thickness of the diffusion layer is thicker.

The formation of SVF is mainly affected by rotation speed [15]. The rotation speed affects the flow field around the electrode surface and changes the distribution of the diffusion layer, which leads to the different etching rates in different areas of the electrode surface. In addition, the etching rate is also related to machining voltage. At a suitable processing voltage, the etching rates in different regions differs greatly, and the surface of the electrode could be machined into a deep spiral groove. To machine deep spiral grooves on the surface of electrodes, it is necessary to combine the speed parameters with the current parameters, that is, the corresponding machining voltages is needed at different rotation speeds to make the cylindrical electrodes into spiral cylindrical electrodes. And strict control of machining time is necessary. When the machining time is short, the spiral groove depth is shallow. When the spiral groove appears on the surface of the cylindrical electrode, the surface of the electrode is no longer a smooth cylindrical surface as the machining proceeds, the SVF on the surface of the electrode will gradually disappear, and the distribution of the diffusion layer will return to its original state. Under the action of tip discharge, the etching rates at the top of the screw threads are higher than that at the bottom of the spiral groove, and the screw threads on the surface of the spiral microelectrode will disappear. Therefore, the main parameters affecting the shape of the spiral cylindrical microelectrode are rotation speed, machining voltage, and time.

## 3. Experiments and discussion

### 3.1. Effect of Rotation Direction on Thread Structure

**Figure 4:**
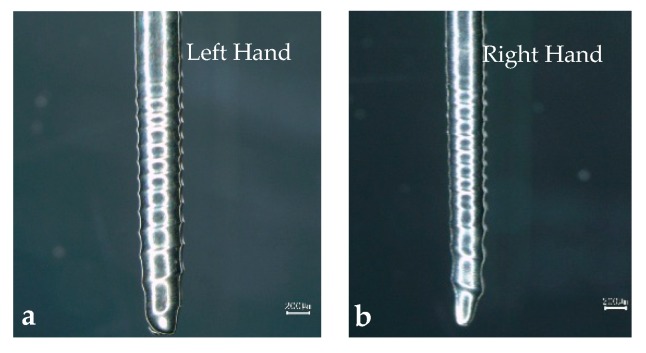
The spiral electrodes fabricated by different rotation directions.

To study the effect of direction on the shape of the spiral cylindrical microelectrode, a set of experiments at different rotation directions were carried out. In Figure 4, compared with Figure 4a,b, it is shown that the revolving directions of the spiral cylindrical microelectrodes are different under different rotation directions. When the electrode rotated clockwise, the cylindrical microelectrode was fabricated into a right-handed spiral cylindrical microelectrode. The cylindrical microelectrode was fabricated into a left-handed spiral cylindrical microelectrode when electrode rotated anti-clockwise, and it is fully illustrated that the flow field around the surface of the microelectrode is one of the main factors affecting the electrode shape. 

### 3.2. Effect of Rotation Speed on Thread Structure

**Figure 5:**
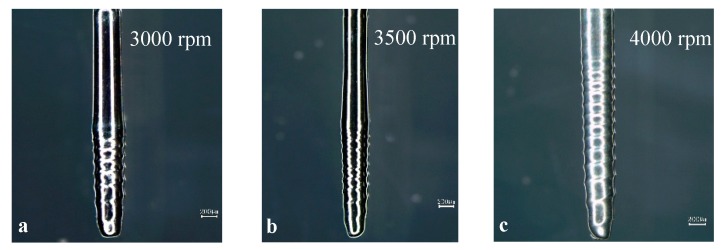
The spiral electrodes fabricated by different rotation speeds.

**Figure 6:**
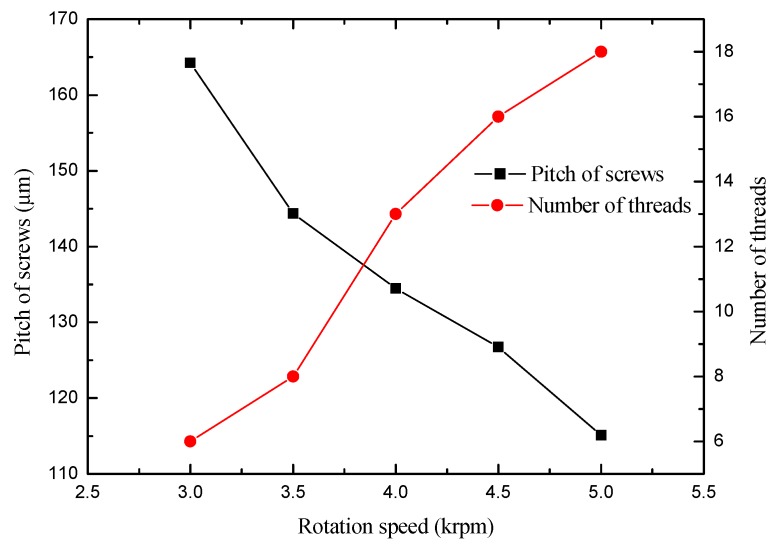
The pitch and the number of screw threads of the spiral electrodes by different rotation speeds.

To study the effect of rotation speed on the shape of the spiral cylindrical microelectrode, a set of experiments at different rotation speeds were carried out. When the initial diameter of the cylindrical tungsten electrode was ∅ 500 μm, the immersion depth was 3 mm, the machining time was 120 s, the rotation speeds were from 3000 to 5000 rpm, and the fabricated spiral cylindrical microelectrodes are shown in Figure 5 and Figure 6.

Figure 5 and Figure 6 show the pitch of the spiral cylindrical microelectrodes and the number of threads at different rotation speeds, the number of threads and pitch on the electrode surface were affected by the rotation speed. When the rotation speed was slow, the threads first appeared at the tip of the microelectrode, and as the rotation speed increased, the number and length of threads increased, and the pitch decreased. The reason for this result is that the rotation speed affected the spiral vortex around the electrode surface. When the rotation speed was low, the spiral vortex was unstable, but the etching rate of the electrode tip was fast, and the larger etching rate difference was easy to form, so the spiral groove on the electrode tip was most easily machined, and this part of the spiral groove was also the deepest. When the rotation speed was large enough, the spiral vortex was more stable, and the area and number of the annular vortices along the axis increased with the increase in the rotation speed. The number and spacing of annular vortices directly affected the number and pitch of the threads on the electrode surface. Within a certain rotation speed range, the faster the rotation speed, then the more the annular vortices around the electrode surface, the smaller the distance between annular vortices, then the longer the length of the fabricated electrode threads, and the more threads, then the smaller pitch.

### 3.3. Effect of Machining Voltage on Depth of Spiral Groove

Compared with the cylindrical microelectrodes fabricated by regular electrochemical etching, the surface of spiral cylindrical microelectrodes fabricated by rotating electrochemical etching was brighter, and the electropolishing reaction occurred on the surface of the spiral microelectrode during the machining process. Therefore, the machining voltage can be determined by the polishing voltage of the spiral cylindrical microelectrode. The polishing voltage is usually determined by the curve of voltage and current density, and the voltage range corresponding to the smooth part of the curve is the electropolishing voltage.

**Figure 7:**
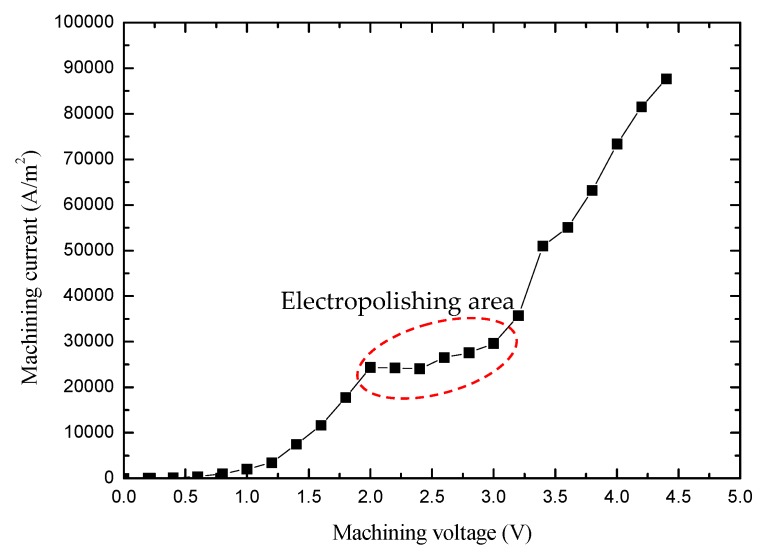
The voltage-current density relation curve.

To determine the electroplating voltage range of the spiral electrode, the anode voltage–current density curve experiment was carried out. When the initial diameter of the cylindrical electrode was ∅ 500 μm, the rotation speed was 4000 rpm, and the immersion depth was 3000 μm. The relation curve of the voltage-current density is shown in Figure 7.

**Figure 8:**
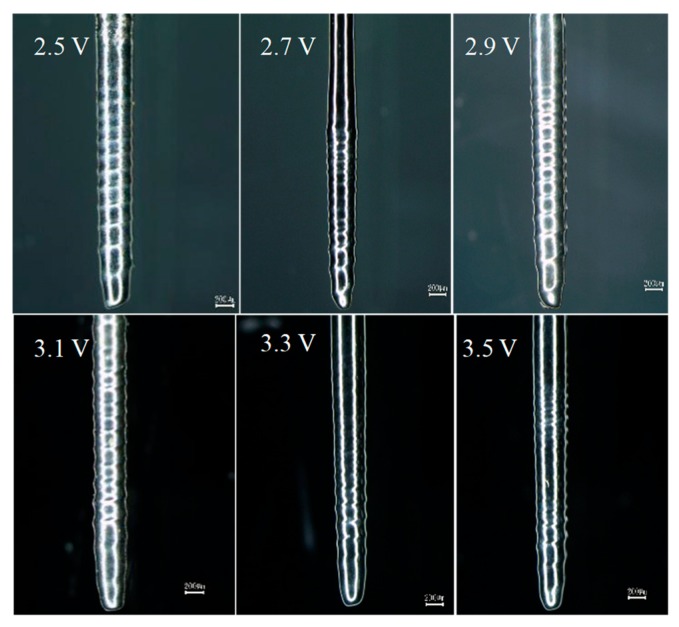
The spiral cylindrical microelectrodes fabricated under different machining voltages.

Figure 7 shows the electropolishing voltage of ∅ 500 μm cylindrical tungsten wire was 2.0 to 3.0 V. To study the effect of electropolishing voltage on the shape of the electrode, six groups of spiral microelectrodes were fabricated by rotating electrochemical etching. When the machining time was 120 s, the machining voltage was 2.5 V, 2.7 V, 2.9 V, 3.1 V, 3.3 V, and 3.5 V, and the other machining conditions were the same as those mentioned above. The experimental results are shown in Figure 8.

Figure 8 shows that the spiral cylindrical microelectrodes can be fabricated by using the electropolishing voltage obtained from the above curve, and the electropolishing reaction occurred on the surface of the spiral microelectrodes. When the machining voltage was lower, the depth of the spiral groove was shallow, and the electrode surface was rough. When the machining voltage was higher, the threads disappeared in some areas of the electrode surface, but the surface brightness was better.

Compared with electrochemical etching, electropolishing is more complicated. Electropolishing caused a more intense passivation reaction on the anode electrode surface, and a dense passivation film was formed, which effectively inhibited the crystallographic corrosion rate of the electrode surface. During the machining process of spiral microelectrodes, the electrolyte in the annular vortex flow renewed quickly, which made the etching rate of the electrode in the annular vortex flow faster. The etching rate appearing on the electrode surface, and the helix of the spiral groove coincided with the SVF. Under normal circumstances, the electric field density at the crest of the thread is larger, and the etching rate is faster than other parts, which is not conducive to the formation of the thread. However, the passivation film produced by electropolishing effectively inhibited the etching rate of the crest. At the same time, the spiral vortex further accelerated the erosion rate of the spiral groove bottom. Therefore, under the combined action of passive film and SVF, the etching rate at the bottom of the spiral groove was faster than that at the crest of thread. The passivation reaction during electropolishing is one of the important conditions for machining the spiral groove.

### 3.4. Effect of Machining Time on Depth of Spiral Groove

During the process of machining spiral cylindrical microelectrodes by rotating electrochemical etching, the machining time is one of the important factors affecting electrode shape. When the machining time is shorter, the depth of the spiral groove is shallower. When the machining time is longer, the SVF is destroyed due to the change in the electrode shape. The etching rate of the thread tip is faster, and the electrode surface gradually becomes cylindrical. 

**Figure 9:**
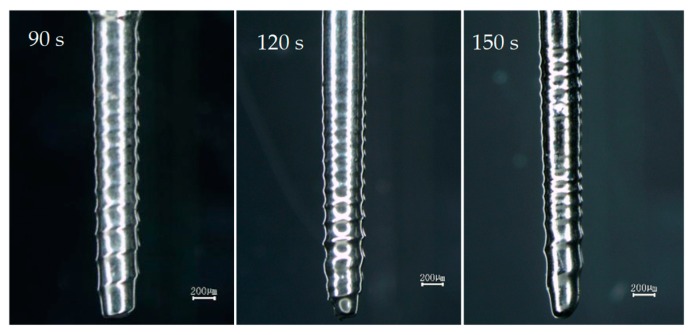
The spiral cylindrical microelectrodes fabricated at different machining times.

To determine the reasonable machining time for spiral cylindrical microelectrodes, three groups of experiments were carried out. When the rotation speed was 4000 rpm, the initial electrode diameter was ∅ 500 μm, the immersion depth was 3 mm, the machining voltage was 2.9 V, and the machining time was 90 s, 120 s, and 150 s. The fabricated spiral cylindrical microelectrodes are shown in Figure 9.

Compared with the microelectrode fabricated in 120 s, the spiral grooves of microelectrodes in 90 s and 150 s were shallower, and part of the thread of the microelectrode fabricated in 120 s disappeared. Therefore, the suitable machining time is in the range of 90 to 150 s, and when the machining time was about 120 s, the shape of the spiral cylindrical microelectrode was better, and the spiral groove was deeper. 

### 3.5. Experimental Results

**Figure 10:**
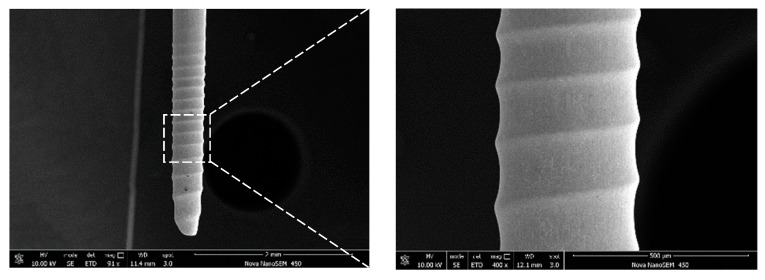
The typical spiral cylindrical microelectrode.

In this paper, a processing technique for machining spiral cylindrical microelectrodes is studied, which has the advantages of high machining efficiency, a small amount of material removal, simple operation, and better shape of the electrode. The spiral cylindrical microelectrodes were fabricated using optimal machining parameters and photographed by Scanning Tunnel Microscope (STM) to demonstrate the machining quality of this process. When the rotation speed was 4000 rpm, the machining voltage was 2.9 V, and the immersion depth was 3 mm. The scanning electron microscopy of the fabricated microelectrodes is shown in Figure 10.

Figure 10 shows that the spiral cylindrical microelectrodes fabricated by using the same optimal machining parameters have obvious spiral grooves and better surface quality. It is demonstrated that rotating electrochemical etching is an effective method to machine spiral grooves on the surface of cylindrical electrodes with short machining time, good surface quality, and high coaxiality.

## 4. Conclusions

In this paper, a new method for machining spiral cylindrical microelectrodes was proposed. The flow field around the electrode surface was analyzed, the effects of rotation speed, rotation direction, machining voltage, and machining time on the surface shape of spiral cylindrical microelectrodes were discussed by experiments. The conclusions can be summarized as follows:

1. The high-speed rotating cylindrical microelectrode causes the SVF, which affects the distribution of the diffusion layer and the mass transfer process around the surface of the microelectrode, and then the spiral groove is formed on the surface of the cylindrical microelectrode due to the velocity difference.
2. The rotation direction of the cylindrical electrode can lead to a left-handed or right-handed spiral electrode. The voltage–current density curve was used to find the appropriate machining voltage within the range of the polishing voltage. Within a certain rotation speed range, the higher the rotational speed, the more the number of threads, the longer the axial coverage range of the threads, and the smaller the pitch of the threads. The machining time directly affects the spiral groove depth on the surface of the microelectrode. Only by choosing the appropriate machining time will an obvious threaded structure be formed on the electrode surface.
3. For further research, in combination with other machining techniques, more shaped electrodes can be fabricated, such as spherical spiral electrodes and disc spiral electrodes.
